# Stressors faced by healthcare professionals and coping strategies during the early stage of the COVID-19 pandemic in Germany

**DOI:** 10.1371/journal.pone.0261502

**Published:** 2022-01-18

**Authors:** Marie Ottilie Frenkel, Katja Mareike Pollak, Oliver Schilling, Laura Voigt, Benedikt Fritzsching, Cornelia Wrzus, Sebastian Egger-Lampl, Uta Merle, Markus Alexander Weigand, Stefan Mohr

**Affiliations:** 1 Institute for Sport and Sport Sciences, Heidelberg University, Heidelberg, Germany; 2 Department of Psychology, University of Freiburg, Freiburg, Germany; 3 Psychological Institute, Heidelberg University, Heidelberg, Germany; 4 Institute of Psychology, German Sport University Cologne, Cologne, Germany; 5 Corona specialized physician practice, Heidelberg, Germany; 6 Head of outpatient service for pediatric patients with fever, Heidelberg, Germany; 7 Center for Technology Experience, Austrian Institute of Technology (AIT) GmbH, Seibersdorf, Austria; 8 Department for Gastroenterology, Infectious diseases and Intoxication, University Hospital Heidelberg, Germany; 9 Department of Anesthesiology, University Hospital Heidelberg, Heidelberg, Germany; University of Auckland, NEW ZEALAND

## Abstract

**Background:**

The COVID-19 pandemic has exerted great pressure on national health systems, which have aimed to ensure comprehensive healthcare at all times. Healthcare professionals working with COVID-19 patients are on the frontline and thereby confronted with enormous demands. Although early reports exist on the psychological impact of the pandemic on frontline medical staff working in Asia, little is known about its impact on healthcare professionals in other countries and across various work sectors. The present cross-sectional, online survey sought to investigate common work stressors among healthcare professionals, their psychological stress as well as coping resources during the pandemic.

**Methods:**

A sample of 575 healthcare professionals (57% male) in three different sectors (hospital, prehospital emergency care, and outpatient service) reported their experiences concerning work and private stressors, psychological stress, and coping strategies between April 17, 2020 and June 5, 2020. To capture pandemic-specific answers, most of the items were adapted or newly developed. Exploratory factor analyses (EFA) were conducted to detect underlying latent factors relating to COVID-specific work stressors. In a next step, the effects of these latent stressors across various work sectors on psychological stress (perceived stress, fatigue, and mood) were examined by means of structural equation models (SEM). To add lived experience to the findings, responses to open-ended questions about healthcare professionals’ stressors, effective crisis measures and prevention, and individual coping strategies were coded inductively, and emergent themes were identified.

**Results:**

The EFA revealed that the examined work stressors can be grouped into four latent factors: “fear of transmission”, “interference of workload with private life”, “uncertainty/lack of knowledge”, and “concerns about the team”. The SEM results showed that “interference of workload with private life” represented the pivotal predictor of psychological stress. “Concerns about the team” had stress-reducing effects. The latent stressors had an equal effect on psychological stress across work sectors. On average, psychological stress levels were moderate, yet differed significantly between sectors (all *p* < .001); the outpatient group experienced reduced calmness and more stress than the other two sectors, while the prehospital group reported lower fatigue than the other two sectors. The prehospital group reported significantly higher concerns about the team than the hospital group (*p* < .001). In their reports, healthcare professionals highlighted regulations such as social distancing and the use of compulsory masks, training, experience and knowledge exchange, and social support as effective coping strategies during the pandemic. The hospital group mainly mentioned organizational measures such as visiting bans as effective crisis measures, whereas the prehospital sector most frequently named governmental measures such as contact restrictions.

**Conclusion:**

The study demonstrated the need for sector-specific crisis measures to effectively address the specific work stressors faced by the outpatient sector in particular. The results on pandemic-specific work stressors reveal that healthcare professionals might benefit from coping strategies that facilitate the utilization of social support. At the workplace, team commitment and knowledge exchange might buffer against adverse psychological stress responses. Schedules during pandemics should give healthcare workers the opportunity to interact with families and friends in ways that facilitate social support outside work. Future studies should investigate cross-sector stressors using a longitudinal design to identify both sector- and time-specific measures. Ultimately, an international comparison of stressors and measures in different sectors of healthcare systems is desirable.

## Introduction

Globally, the COVID-19 pandemic has posed major challenges for public health systems. Besides stretching the capacities of intensive care units (ICU), healthcare professionals have represented the most critical resource for saving lives and limiting the impact of the pandemic [1]. As known from previous pandemic studies [2–4], healthcare professionals experience great psychological stress, while still being expected to act functionally at work. By acknowledging the need for constant healthcare provisions throughout the pandemic, effective crisis management that is targeted at reducing healthcare professionals’ psychological stress is required to protect their mental health, well-being, and functioning [5]. In view of this, the present study investigated COVID-19-specific work stressors, psychological stress, and coping resources among healthcare professionals, with the aim of improving mental health leaders’ and policy makers’ understanding of effective crisis management in pandemics.

According to the transactional model of stress [6], a perceived discrepancy between the environmental demands placed on an individual and their available coping resources leads to stress. If environmental demands are perceived as threatening (i.e., stressor), an individual evaluates their available coping resources to determine whether they feel able to cope with the stressor. In times of heightened demands, such as during a pandemic, effective crisis prevention (taken before the stressful event), crisis measures (taken during the stressful event), as well as individual coping resources can be perceived as helpful to overcome the stressor, resulting in a "challenge" or neutral appraisal of the situation. However, if coping resources turn out to be inadequate, an individual experiences a negative psychological state of stress, which can include an increase of perceived stress and mood deteriorations [7].

Previous studies conducted during the outbreaks of SARS and Ebola have already shed some light on stressors that healthcare professionals have to face during pandemics. The major work stressors found in these studies included the feeling of risk towards getting infected and the fear of infecting one’s family, friends, and colleagues [8], worries arising due to uncertainty and stigmatization [8, 9] and a reluctance to work or contemplation of resignation [9]. Healthcare professionals reported high levels of work stress and reduced well-being [8–10], which were found to have long-term implications for their mental health [11–13]. Similar concerns have been raised about workload and its effects on the stress levels of healthcare professionals who are responsible for the treatment of patients with COVID-19 [1, 4, 14–18]. As a result, in recent months, several health organizations [19–21] (Inter-Agency Standing Committee, International Federation of Red Cross and Red Crescent Societies, World Health Organization) and research groups [4, 5, 14, 22, 23] have discussed a variety of COVID-19-specific interventions to support healthcare professionals’ ability to cope with their daily work. As a first step, the Inter-Agency Standing Committee provided a list of COVID-19-specific stressors of frontline healthcare professionals. Examples of items on this list were “strict bio-security measures”, “insufficient personal or energy capacity to implement basic self-care”, and “[the] fear that healthcare workers will pass COVID-19 onto their friends and family as a result of their work”. It is striking that, to date, almost all empirical studies on psychological stress during the ongoing pandemic focus on the effects of stress and fail to take a closer look at the stressors. Only one study integrated a selection of stressors at work [15], using an adapted short questionnaire on stressors during the SARS pandemic [24]. In summary, there is no comprehensive list of stressors formulated within current research that makes use of empirical data, as proposed by the Inter-Agency Standing Committee.

Despite all the potential stressors, the availability of coping resources will ultimately determine whether the stressors lead to high stress levels [6]. While the risk of infection with COVID-19 is considered stressful in the general population [25] and among other frontline workers [1, 23, 26], healthcare professionals are well-trained in dealing with infectious patients, which might increase their perception of available coping resources [27–29]. Additional coping resources in the current pandemic might be governmental and organizational COVID-19 procedures, such as visiting bans in hospitals [30]. However, it is unclear which of these measures are perceived as effective in reducing psychological stress at work by healthcare professionals. This constitutes a further research gap addressed by this study.

Due to the differential spread of COVID-19, countries have been affected more or less severely at different times. Thus, research focused on different countries in the beginning. A review of 14 empirical studies on the pandemic-related stress of healthcare professionals, published in the early stage of the COVID-19 pandemic from January to March 2020, revealed that the integrated studies only investigated individuals working in Asia, without extending attention to healthcare professionals’ work stressors during COVID-19 to the rest of the world [4]. National health systems, their preparedness for a pandemic as well as their capacities to deal with increased demands differ greatly between countries. For example, Germany was relatively well prepared due to early warnings from other countries and higher ICU capacities per capita. Additionally, the outpatient care sector through general practitioners and specialists in private practice represents an important pillar of the German healthcare system [4]. The outpatient care sector has emerged as a major factor for Germany’s strong enabling environment within the COVID-19 pandemic. However, previous studies focused on the comparison within different clinical departments and occupations in hospitals [4, 23]. Therefore, it remains unclear whether different sectors (i.e., hospital, prehospital emergency care, and outpatient sector) experience different stressors, perceive different coping resources to be effective, and are thus differently stressed.

The overall aim of the present study was to identify target group-specific work stressors and coping resources to inform best practices in crisis management for further waves of COVID-19 or future outbreaks of pandemics. Building on existing assumptions of COVID-19 work-specific stressors [19], the study aimed to quantify common work stressors among healthcare professionals, assess their impact on professionals’ psychological stress and identify effective coping resources to counteract stressors by comparing hospital, prehospital emergency care and outpatient sectors.

## Methods

The prospective repeated cross-sectional, observational study was conducted in Germany during the early stage of the COVID-19 pandemic from April 17, 2020 to June 5, 2020. The study was originally designed as a longitudinal survey to be administered at three different time points during the pandemic (April 17–24; May 8–15; May 29—June 5, 2020). However, the repeated recruitment of participants proved more difficult than expected due to low response rates in follow-up surveys. As a result, we treated the data as cross-sectional and do not report changes over time.

In mid-March, the federal states [5] started to close daycare centres, kindergartens and schools. On March 23, 2020, the federal states and the national government [5] implemented a “contact restriction” measure, limiting public gatherings to two people (outside families), enforcing physical distance of at least five feet (1.5 meters), and closing many businesses. A gradual easing of physical distancing followed during our last measurement. The wearing of masks was only recommended in public during the first measurement and became obligatory during our second and third measurement.

### Sample and procedure

The sample consisted of *N* = 575 healthcare professionals. To examine a large sample of healthcare staff from the hospital, prehospital emergency care and outpatient sectors, the survey was distributed online with the software SoSci Survey (http://www.soscisurvey.de). Participants were recruited by asking the medical directors of hospitals to forward the study invitation to staff and to post it on social media (e.g., medical forums, newspapers, Facebook groups, and other channels such as medical blogs and mailing lists).

The Ethics Committee of the Faculty of Behavioral and Cultural Studies, Heidelberg University, Germany provided ethical approval for this study (approval number: AZ Fre 2020 1/1). Informed written consent was obtained from the participants. Participants received no financial compensation.

### Measures

The survey length was kept as short as possible in order to ensure high participation rates and to minimize interference with professional duties. This was achieved by using a questionnaire that was originally designed for ecological momentary assessment [31], a list of COVID-19 related stressors for healthcare professionals [19] and several self-developed items, which were successfully used in previous studies [26, 32–34]. Explorative data was gained from six additional open-ended questions [see 26, S1 Table, column 2, 35], allowing participants to share their variety of ongoing experiences. On average, participants took 10–12 minutes to complete the questionnaire.

Private stressors were assessed by asking for extraordinary private demands (e.g., whether one had caught COVID-19, death of a relative, ongoing divorce) [19]. Furthermore, participants were asked to rate the perceived stressfulness of 19 work stressors (e.g., “fear of getting infected”, “fear of infecting others”) on a scale ranging from 1 (*not at all*) to 7 (*very*). Potential stressors (see first column of Table 1) were adapted from a list of stressors for healthcare professionals in the early stages of the COVID-19 pandemic [19]. Participants could name additional work stressors in an open-ended question.

**Table 1:** Means, standard deviations and bivariate correlations of different stressors.

	Stressors	M	SD	1	2	3	4	5	6	7	8	9	10	11	12	13	14	15	16	17	18	19
1	“Fear of getting infected”	3.29	1.81	–																		
2	“Fear of infecting others”	4.31	1.96	.47	–																	
3	“Worrying about family members/children being at home”	4.05	2.08	.34	.40	–																
4	“Worries about health deterioration of vulnerable family members and friends”	4.83	1.77	.37	.56	.55	–															
5	“Stigmatization”	3.33	1.90	.35	.32	.26	.38	–														
6	“Strict bio-security measures”	5.00	1.78	.24	.19	.26	.24	.27	–													
7	“Higher work demands”	4.32	1.87	.32	.31	.33	.33	.34	.43	–												
8	“Reduced capacity to use social support”	3.92	2.01	.29	.28	.32	.34	.60	.41	.54	–											
9	“Insufficient capacity to implement basic self-care”	4.01	1.86	.26	.27	.35	.36	.37	.35	.53	**.60**	–										
10	“Insufficient information about long-term exposure to infected individuals”	3.84	1.98	.45	.45	.35	.44	.45	.36	.37	.48	.44	–									
11	“Fear of infecting friends and family”	4.63	1.98	.51	**.74**	.52	.64	.39	.31	.40	.36	.37	.51	–								
12	“Confrontation by patients who fear anger against the government”	3.82	1.93	.26	.27	.32	.40	.38	.27	.33	.45	.43	.44	.37	–							
13	“Information overload of constantly changing information”	4.87	1.81	.23	.21	.25	.27	.28	.40	.32	.35	.37	.42	.28	.40	–						
14	“No clear instructions”	4.73	1.93	.31	.33	.30	.34	.35	.35	.32	.41	.42	.56	.41	.43	**.66**	–					
15	“Fear of being isolated from usual work team”	3.24	1.89	.22	.21	.23	.24	**.42**	.30	.35	.50	.38	.38	.28	.31	.33	.40	–				
16	“Worries about heavier workload of coworkers when falling ill”	3.47	1.98	.34	.36	.31	.35	.37	.24	.32	.38	.38	.37	.39	.34	.30	.33	.44	–			
17	“Insufficient protective clothing”	4.34	2.19	.37	.30	.24	.27	.26	.22	.26	.29	.30	.44	.36	.35	.31	.47	.26	.26	–		
18	“Difficult reconciliation of work and family”	3.90	2.06	.36	.34	.46	.39	.39	.34	.43	.51	.60	.47	.43	.43	.35	.45	.39	.42	.39	–	
19	“Fear of passing virus to workplace”	3.20	1.96	.42	.52	.31	.39	.36	.23	.34	.28	.26	.41	.52	.28	.22	.37	.34	.48	.32	.36	–

Note. The Likert-Scale ranged from 1 (*not at all*) to 7 (*very*). All correlations are significant on a level of *p* < .001. Correlations are highlighted between two items with the highest loadings on the respective factor identified in the exploratory factor analysis.

#### Psychological stress

Actual perceived stress was measured using the single item “during the last week, I felt stressed out” which was rated from 1 (*not at all*) to 5 (*very*) [26, 32–34]. Fatigue was measured using the single item “during the last week, I felt fatigued” rated from 1 (*not at all*) to 5 (*very*) [26, 32–34].

*Mood* was measured by a six-item short version of the German Multidimensional Mood Questionnaire [31]. Three bipolar scales represent *valence* (V), *energy* (E) and *calmness* (C) [content–discontent (V–), tired–awake (E+), full of energy–without energy (E–), unwell–well (V+), agitated–calm (C+), relaxed–tense (C–)]. Each item was rated on a five-point scale ranging from 1 (*not at all*) to 5 (*very*). Wilhelm and Schoebi [31] reported good structural validity, sensitivity to change and reliability of this short scale. For the analyses, data from three items (i.e., V–, E–, C–) were reverse-coded. For V (α = .75), E (α = .79), and C (α = .74), average scores were calculated.

#### Crisis management

After having indicated whether their area of work or function had changed through the COVID-19 pandemic, participants were asked to name effective crisis measures taken by the government, and/or the health system during the pandemic [see 26, S1 Table, second column]. In addition, participants were asked to rate their satisfaction with the measures on a scale ranging from 1 (*not at all*) to 7 (*very*). Furthermore, participants were asked in an open-ended question to name effective crisis prevention measures before the pandemic, i.e., those measures that have prepared them for the work demands during the current pandemic [26]. Again, participants were asked to rate how well their education and/or training prepared them for the current work demands during the COVID-19 crisis on a scale ranging from 1 (*not at all*) to 7 (*very*) [26]. Finally, participants were asked in an open-ended question to list their individual coping strategies.

### Data analysis

#### Latent variable analyses

Exploratory factor analyses (EFA) were conducted to account for the intercorrelations between the 19 work stressors, examining the underlying latent factors of COVID-specific stressful experiences at work. In particular, we ran Maximum Likelihood EFA with Mplus 8.1, using the MLR estimator (to account for potential data non-normality) and modelling a “complex” data structure which accounts for dependencies of multiple observations from some respondents (for details see [36]). The Bayes Information Criterion (BIC) served as a decision criterium of the number of factors. We ran models with 1 to 9 factors and selected the solution which minimized the BIC [37]. *Geomin* rotation was used to allow for correlations between the factors.

**Fig 1:**
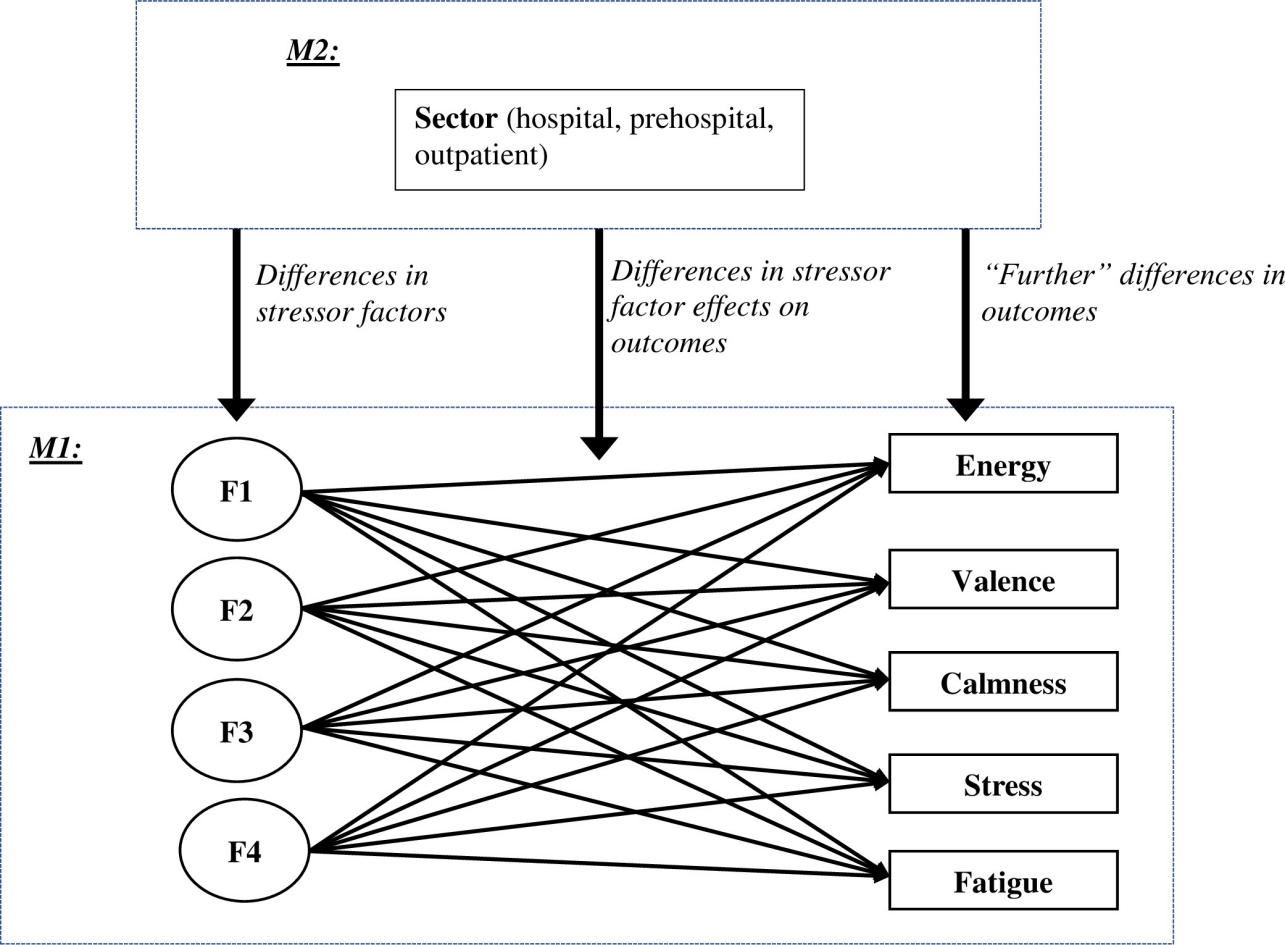
Structural equation modeling: Analysis plan. F = factor, F1 = fear of transmission, F2 = interference of workload with private life, F3 = uncertainty/lack of knowledge, F4 = concerns about the team. *M1 = Overall Model*, *M2 = Work Sector*.

Finally, we included the solution obtained from the EFA in structural equation models (SEM) to examine the effects of the latent work stress factors on psychological stress. The chosen statistical method allows variability to be described among the observed correlated stressors in terms of a potentially lower number of latent (unobserved) variables called stress factors. Furthermore, we checked whether different work sectors affected latent work stress factors and psychological stress. We ran two SEM, as illustrated in Fig 1 (again using the MLR estimator and modelling a “complex” data structure). Notably, the four latent factors obtained from the EFA step were included in these SEM by keeping the 4×19 (unstandardized) factor loadings, as well as the 19 work stressor intercepts estimated for the EFA solution, fixed in all these analyses. First, we modelled the observed scores of five psychological stress outcomes (i.e., perceived stress, fatigue, energy, valence, and calmness) predicted by the latent work stress factors (M1). Second, we analyzed work-sector-related differences by means of a multigroup SEM (M2) and Wald tests of parameter constraints [36], assessing whether the path coefficients of different sectors could be equated without significant loss of model fit. We also ran both models controlling for age and sex i.e., both were added as predictors for the outcome, which did not result in any notable changes in the results reported below (regarding neither values nor the significance of the estimated coefficients). Therefore, for reasons of space, we present the results of the “uncontrolled” analyses below.

#### Qualitative analyses

The open-ended questions were analyzed and coded on the basis of the deductive category assignment in the qualitative content analysis according to Mayring [38]. Quotations were derived from the answers to the open-ended questions and served as data units in the analysis. Each data unit consists of an independently interpretable and meaningful unit. When a participant’s quotation addressed more than one meaningful issue, it was divided into multiple data units. For example, when a respondent listed three additional work demands, this answer was split into three data units, each listing one work demand. For the coding system, main categories and subcategories were derived inductively for each question based on the material from approximately 50% of the dataset. All data units were assigned to the existing main categories. If a data unit could not be clearly assigned to any of the defined subcategories, it was sorted into a main category. These frequencies were used to identify main themes, changes or differences between sectors, which are in turn described in more detail by providing individual quotations. We reported an overall response rate for each open-ended question, which refers to the percentage of surveys that have covered the respective open-ended question. It should be noted that, due to the repeated participation of few participants, the overall response rate might differ slightly from the percentage of participants that have answered the respective question.

## Results

### Sample description

A total of *N* = 615 observations were used for the analyses. Notably, these observations were obtained from 575 participants (57% male), including eight individuals (1.4%) who participated at all three measurement points and 24 individuals (4.2%) who participated twice. Thus, a total of 543 (94.4%) persons participated only at one of three measurement points, thereby delivering no “truly longitudinal” information on intra-individual change. The participants’ age ranged from <20 to 65–69 years. More than half of the participants (58.4%) were aged between 25 and 44 years. Participants’ work experience ranged from 0 to more than 40 years.

In all sectors, roughly half of the participants reported a change of their work area or function due to the pandemic (55% in the hospital sector, 44% in the prehospital sector, and 48% in the outpatient sector). The subsample from the hospital sector (*n* = 254) included medical doctors, nurses, and undergraduate medical students. The prehospital subsample (*n* = 250) comprised emergency physicians and paramedics. Regarding the outpatient sector (*n* = 108), participants were medical doctors, undergraduate medical students, and medical assistants working at general, family or pediatric practices which specialized in the treatment of COVID-19 patients. Three participants did not provide any information about their work sector and were thus excluded from the analysis which focused on differences between work sectors.

### Private stressors during the pandemic

Most of the healthcare professionals did not report private stressors (overall response rate of 29.3%). One third of the given answers was related to the main subject “worries about the health of relatives”. “Caregiving duties” was the second most frequently mentioned theme (S3 Table). In relation to the first main subject (worries about relatives), healthcare professionals mostly mentioned not only relatives and/or friends who belonged to certain risk groups but also stigmatization from family members and friends who were afraid of getting infected. A few participants reported extreme accumulation of family duties or critical life events, such as the dead of a relative or friend.

– *Wife is afraid of being infected by me*. *Constant domestic discussions about the dangerous nature of the situation*.

*(male*, *65–69 years old*, *prehospital sector)*

In relation to the second main subject (caregiving duties), participants most frequently mentioned childcare and home schooling as stressful caregiving duties.

– *Childcare with 2 full-time working parents with very limited home office facilities*. *(female*, *40–44 years old*, *prehospital sector)*

### Work stressors during the pandemic

Regarding work-related stressors, participants’ ratings on the perceived stressfulness of the 19 pandemic-specific stressors (Table 1) revealed that the necessity of having “strict safety measures” was considered as most stressful. “Fear of getting infected” and “feeling of being isolated from usual work team” were comparably low work stressors.

To detect groups of stressors that belong together or co-occurred frequently, we conducted an exploratory factor analyses (EFA) of the 19 distinct stressors. Intercorrelations between the 19 different stressors are presented in Table 1. The EFA revealed four distinct factors (i.e., groups of stressors) because the 4-factorial model demonstrated the best fit with the lowest information criterion BIC (BICs for models with 1 to 9 factors, respectively: 45040.5, 44482.4, 44303.5, 44280.3, 44287.8, 44309.2, 44330.0, 44367.1, 44406.6). In terms of widely used fit indices, this 4-factor model provided good to acceptable fit to the data (RMSEA = 0.06, CFI = 0.947, SRMR = 0.027). Hence, four factors were extracted in total (S1 Table for factor correlations, and S2 Table for factor loadings). In interpreting the core items with highest loadings/factor correlations for each factor (S2 Table), the first factor can be characterized as “fear of transmission” (core items: “fear of infecting others”, λ = 0.898; “fear of infecting friends and family”, λ = 0.881), the second as “interference of workload with private life” (core items: “reduced capacity to use social support due to long work time and stigmatization of healthcare workers”, λ = 0.826; “insufficient capacity to implement basic self-care due to lack of time and energy”, λ = 0.734), and the third as “uncertainty/lack of knowledge” (core items: “information overload of constantly changing information”, λ = 0.740; “no clear instructions”, λ = 0.909).

The significance of the fourth factor seems less clear. Whereas even the items with relatively high loadings on this factor show higher loadings on one of the other factors, this fourth factor revealed the lowest correlations with the others. This suggests that this fourth factor reflects some additional source of variance which is largely independent of the other factors, which affects some items that mainly “belong” to one of the first three factors. Notably, the wording of all items loading relatively high on this factor have in common a notion of working within a team (e.g., highest loading core items: “feeling isolated due to separation from usual work team”, λ = 0.339; “stigmatization of oneself and other healthcare personnel working with COVID-19 infected patients”, λ = 0.311). The factor comprises stressors that are likely to affect the respondent more, the more they feel integrated into the work team. The work teams consist of colleagues in the healthcare sector in which the respondents work. Therefore, this factor may be interpreted in terms of “concerns about the team”, a factor which may include both additional stress in terms of responsibility for the team and resilience due to increased team integration.

Additional work stressors which were asked about through an open-ended question were mentioned in only 20% of the surveys. “Non-compliance of the society” (12%), “medical supply shortage” (11%) and “increased workload” (11%) were mentioned most frequently.

### Psychological stress

The results showed that “interference of workload with private life” was the pivotal predictor of psychological stress, whereas “concerns about the team” had stress-reducing effects. Generally, stressors had equal effects on psychological stress across work sectors. We now present the results in detail.

Results from the structural equation models M1 and M2 (estimates of intercepts and path coefficients) are summarized in Table 2 (see Fig 1 for an illustration of the models) below.

**Table 2:** Structural equation model results: estimates of factor means, intercepts, and path coefficients from models 1 and 2.

	Overall model (M1)			Multigroup model: sector (M3)								
				Hospital sector			Prehospital sector			Outpatient sector		
	Est.	SE	*p*	Est.	SE	*p*	Est.	SE	*p*	Est.	SE	*p*
*Means*:												
F1	0	Fixed		0	fixed		0.079 [-.034, .192]	0.069	.249	0.035 [-.136, .205]	0.104	.736
F2	0	Fixed		0	fixed		-.057 [-.173, .059]	0.070	.417	0.084 [-.095, .262]	0.109	.440
F3	0	Fixed		0	fixed		-.068 [-.183, .048]	0.070	.335	0.128 [-.039, .294]	0.101	.207
F4	0	Fixed		0	fixed		**0.344 [.193, .496]**	0.092	< .001	-.087 [-.303, .129]	0.132	.508
*Intercepts*:												
Energy	2.750 [2.691, 2.808]	0.036	< .001	2.717 [2.625, 2.810]	0.056	< .001	2.854 [2.753, 2.954]	0.061	< .001	2.599 [2.435, 2.763]	0.100	< .001
Valence	3.103 [3.045, 3.162]	0.036	< .001	3.101 [3.007, 3.195]	0.057	< .001	3.192 [3.080, 3.304]	0.068	< .001	2.920 [2.781, 3.058]	0.084	< .001
Calmness	3.015 [2.954, 3.075]	0.037	< .001	3.031 [2.945, 3.117]	0.052	< .001	3.174 [3.064, 3.285]	0.067	< .001	2.700 [2.531, 2.869]	0.103	< .001
Stress	3.228 [3.167, 3.289]	0.037	< .001	3.184 [3.100, 3.268]	0.051	< .001	3.083 [2.977, 3.189]	0.064	< .001	3.488 [3.309, 3.667]	0.109	< .001
Fatigue	3.273 [3.212, 3.334]	0.037	< .001	3.342 [3.250, 3.435]	0.056	< .001	3.077 [2.966, 3.188]	0.067	< .001	3.475 [3.317, 3.633]	0.096	< .001
*Path coefficients*:												
Energy:												
F1 ➨	-.046 [-.127, .035]	0.049	.349	-.057 [-.169, .055]	0.068	.401	-.112 [-.231, .008]	0.073	.123	0.048 [-.195, .290]	0.148	.746
F2 ➨	**-.513 [-.612, -.414]**	0.060	< .001	**-.485 [-.638, -.332]**	0.093	< .001	**-.520 [-.659, -.382]**	0.084	< .001	**-.577 [-.868, -.287]**	0.177	.001
F3 ➨	-.069 [-.169, .030]	0.061	.252	-.146 [-.305, .013]	0.097	.131	0.019 [-.117, .155]	0.083	.816	0.028 [-.222, .278]	0.152	.854
F4 ➨	**0.219 [.126, .312]**	0.056	< .001	**0.273 [.145, .401]**	0.078	< .001	0.089 [-.048, .225]	0.083	.286	**0.428 [.197, .660]**	0.140	.002
Valence:												
F1 ➨	-.041 [-.120, .038]	0.048	.397	-.018 [-.125, .090]	0.066	.790	-.127 [-.269, .016]	0.086	.143	0.041 [-.169, .250]	0.127	.749
F2 ➨	**-.380 [-.475, -.286]**	0.057	< .001	**-.340 [-.494, -.186]**	0.093	< .001	**-.396 [-.537, -.254]**	0.086	< .001	**-.456 [-.699, -.213]**	0.148	.002
F3 ➨	**-.149 [-.245, -.053]**	0.058	.011	**-.198 [-.362, -.035]**	0.100	.046	-.040 [-.180, .100]	0.085	.640	-.145 [-.372, .082]	0.138	.294
F4 ➨	0.090 [-.001, .182]	0.056	.103	0.034 [-.110, .178]	0.087	.696	0.025 [-.126, .177]	0.092	.782	**0.306 [.095, .516]**	0.128	.017
Calmness:												
F1 ➨	-.052 [-.135, .031]	0.050	.300	0.021 [-.086, .127]	0.065	.749	-.159 [-.298, -.021]	0.084	.058	-.080 [-.308, .149]	0.139	.566
F2 ➨	**-.440 [-.537, -.343]**	0.059	< .001	**-.487 [-.622, -.352]**	0.082	< .001	**-.395 [-.545, -.245]**	0.091	< .001	**-.371 [-.635, -.107]**	0.161	.021
F3 ➨	**-.140 [-.237, -.043]**	0.059	.017	**-.205 [-.349, -.061]**	0.088	.019	-.023 [-.161, .115]	0.084	.784	-.087 [-.334, .160]	0.150	.563
F4 ➨	**0.161 [.068, .255]**	0.057	.005	**0.187 [.051, .323]**	0.083	.023	0.012 [-.130, .155]	0.087	.887	**0.344 [.147, .542]**	0.120	.004
Stress:												
F1 ➨	0.089 [.004, .174]	0.052	.085	0.030 [-.068, .128]	0.060	.615	**0.247 [.117, .376]**	0.079	.002	-.066 [-.326, .193]	0.158	.674
F2 ➨	**0.532 [.432, .632]**	0.061	< .001	**0.560 [.439, .681]**	0.074	< .001	**0.421 [.263, .579]**	0.096	< .001	**0.699 [.398, 1.000]**	0.183	.000
F3 ➨	0.082 [-.012, .176]	0.057	.149	**0.176 [.054, .298]**	0.074	.018	-.084 [-.230, .062]	0.089	.342	0.097 [-.185, .379]	0.172	.571
F4 ➨	**-.153 [-.247, -.060]**	0.057	.007	**-.280 [-.409, -.150]**	0.079	< .001	0.034 [-.102, .171]	0.083	.681	**-.305 [-.542, -.068]**	0.144	.034
Fatigue:												
F1 ➨	0.051 [-.028, .130]	0.048	.291	0.071 [-.034, .176]	0.064	.266	0.093 [-.016, .201]	0.066	.160	0.009 [-.214, .233]	0.136	.945
F2 ➨	**0.511 [.413, .609]**	0.060	< .001	**0.496 [.345, .647]**	0.092	< .001	**0.452 [.308, .595]**	0.087	< .001	**0.669 [.406, .931]**	0.159	.000
F3 ➨	0.113 [.017, .210]	0.059	.054	0.130 [-.028, .287]	0.096	.175	-.018 [-.164, .128]	0.089	.839	0.154 [-.071, .379]	0.137	.260
F4 ➨	**-.190 [-.279, -.101]**	0.054	< .001	**-.175 [-.303, -.047]**	0.078	.025	-.022 [-.176, .133]	0.094	.816	**-.372 [-.581, -.164]**	0.127	.003
*Moderation tests*: *between group differences of path coefficients*												
							Wald^b^	Df	*p*			
All ➨^a^							50.993	40	.114			

Note. F = Factor; F1 = fear of transmission, F2 = interference of workload with private life, F3 = uncertainty/lack of knowledge, and F4 = concerns about the team. Numbers in brackets are 95% confidence intervals. ^a^ All path coefficients (outcomes predicted by factors) constrained equally across groups;

^b^ Wald test statistic obtained from Mplus test of parameter constraints.

*Overall Model (M1)*: The factor “fear of transmission” did not reveal any significant effects predicting outcomes of energy, valence, calmness, stress, and fatigue (all *p*s > .09). The factor "uncertainty/lack of knowledge" showed only small effects predicting these outcomes, with absolute coefficient values ranging from 0.07 to 0.15 (although it was significant at *p* < .05 in predicting valence and calmness). The factor “interference of workload with private life” was found to be the pivotal factor in predicting low energy (b = -0.51, *p* < .001), valence (b = -0.38, *p* < .001), calmness (b = -0.44, *p* < .001), and high levels of stress (b = 0.53, *p* < .001), and fatigue (b = 0.51, *p* < .001). In contrast, the factor “concerns about the team” showed some smaller effects, predicting higher energy (b = 0.219, *p* < .001) and calmness (b = 0.161, *p* < .01) and less stress (b = -0.153, *p* < .01) and fatigue (b = -0.190, *p* < .001). M1 fitted the data well (RMSEA = 0.035, CFI = 0.967, SRMR = 0.057).

*Work Sector (M2)*: The overall test for the equality of all coefficients across the three sectors did not show a significant result (W[40] = 50.99, *p* = .11), indicating that between-sector differences in the path coefficients could be constrained equally without loss of model fit. Thus, the effects of the identified factors on the outcomes did not differ substantially between the work sectors. Regarding the factor means, the factor “concerns about the team” was significantly higher in the prehospital sector than in the hospital sector. There were no other between-sector mean differences. Notably, however, the outcome intercepts differed between the sectors (overall Wald test: W[10] = 23.22, *p* = .01). Further tests on each of the outcomes showed that these between-sector differences are significant for calmness (W[2] = 15.01, *p* < .001), stress (W[2] = 10.30, *p* = .006) and fatigue (W[2] = 14.37, *p* < .001), and without α-adjustment for multiple testing also for valence (W[2] = 6.34, *p* = .04). Thus, as intercept differences could be taken as an indicator of direct sector effects on the outcomes (i.e., effects which are unmediated by the stressor factors), respondents from the outpatient group experienced reduced calmness and felt more stressed, whereas the prehospital group reported lower fatigue. M2 fitted slightly worse than M1, but still fairly well (RMSEA = 0.047, CFI = 0.942, SRMR = 0.065).

### Crisis management

Regarding crisis management, healthcare professionals highlighted social distancing and compulsory masks, training, experience and knowledge exchange, and social support as effective coping strategies during the pandemic. The hospital group mainly mentioned organizational measures such as visiting bans as effective crisis measures, whereas the prehospital sector most frequently named governmental measures such as contact restrictions. We will now proceed to describe the results in detail.

Descriptive data of the satisfaction ratings for crisis measures and satisfaction with crisis prevention are presented in Table 3. Satisfaction with crisis measures and prevention was rated medium across all sectors on average, with means ranging from 4.23 to 4.54 for crisis measures and from 3.64 to 3.71 for crisis prevention. Main categories of themes derived from the open-ended questions and frequencies of quotations are reported for each sector (S3 Table).

**Table 3:** Satisfaction with short-term crisis measures and crisis prevention.

	Satisfaction with crisis measures	Satisfaction with crisis prevention*
Hospital sector	4.54 (1.66)	3.71 (1.87)
Prehospital sector	4.27 (1.79)	3.64 (1.90)
Outpatient sector	4.23 (1.88)	3.67 (1.98)

Note. Means and standard deviations are displayed in brackets. Satisfaction and crises prevention were assessed on a scale ranging from 1 (*not at all*) to 7 (*very*). Descriptive statistics include all data points and do not adjust for the few participants who participated twice and three times (see Methods for details).

*Crisis prevention was assessed by asking participants how well education and training prepared them for the crisis.

#### Effective crisis measures

More than half of the healthcare professionals responded to the open-ended question about subjectively perceived effective crisis measures (57.2%).

On the governmental level, healthcare professionals mainly perceived social distancing regulations and compulsory oronasal masks for the general population as effective.

– *Requirements to keep distance to others create awareness of the gravity of the situation*. *(male*, *50–54*, *hospital sector)*

– *Patients are used to masks*, *which they now must also wear in ambulances and hospitals in case of emergency*. *(male*, *30–34 years old*, *prehospital sector)*

On the organizational level, healthcare professionals from all sectors perceived visiting bans, bans on elective surgery, and supply of protective equipment for healthcare staff as useful.

– *Visiting bans of relatives in hospitals; much quieter surroundings and less drama*

*(male*, *20–24 years old*, *prehospital sector)*

– *No plannable surgeries*, *therefore [there is] more staff on [the] intensive care unit*.

*(female*, *45–49 years old*, *hospital sector)*

It appears that the perceived effectiveness of these regulations varied across sectors. Organizational measures were mostly mentioned in the hospital sector, whereas governmental measures were more often introduced in the prehospital sector.

At the same time, approximately a fifth of the given answers were subsumed under the category “no effective crisis measures have been taken”. In the descriptions of the healthcare professionals, different reasons can be identified for this outcome: a) no measures were taken because no measures were necessary, b) measures should have been taken, but they were not, c) measures were taken but were not sufficient or effective, and d) measures that were taken even aggravated the work.

*a) We have been relatively well prepared by the annual influenza wave*. *(female*, *30–34 years old*, *outpatient sector)*

*b) Nothing at all*. *In my opinion the supply of protective masks and gowns was insufficient*. *(male*, *20–24 years old*, *hospital sector)*

*c) Since unfortunately people are very inconsistent with the “rule”*, *which is not even controlled properly*, *I am very angry*.

*(male*, *20–24 years old*, *sector unknown)*

*d) None! I feel no relief from the crisis measures taken*. *On the contrary*, *my field of activity has expanded*.

*(male*, *55–59 years old*, *hospital sector)*

#### Effective crisis prevention

Half of the healthcare professionals named effective crisis prevention (overall response rate of 51.4%). Healthcare professionals perceived three main factors as effective in crisis prevention (which together made up more than 67% of the answers, S3 Table): (1) aspects of their professional training, (2) general work experience and exchange, and (3) crisis measures implemented at an early stage.

Regarding the training of healthcare professionals, participants mostly mentioned general hygienic training (infection protection courses dealing with highly infectious patients), followed by specific hygienic training related to SARS CoV-2 and further vocational training with a focus on crisis management. In particular, they described repetition and regular updating of such courses as key principles that lead to additional safety when dealing with infectious patients.

– *Re-training on materials and the correct way to put on and take off the infection protection equipment*. *(female*, *20–24 years old*, *prehospital sector)*

– *A bunch of good training courses held by the employer of the clinic*, *e*.*g*., *a course related to protective clothing as well as additional information via e-mails I received regularly*. *(female*, *60–64 years old*, *hospital sector)*

Besides training, healthcare professionals reported work experience and sharing current work experiences with colleagues as an effective resource during the pandemic. Having experience in dealing with highly infectious patients was perceived as helpful. Occasionally, participants reported that experiences and lessons learnt from other viruses (e.g., avian influenza), other pandemics (e.g., swine flu pandemic) or similar contexts (civil protection activities) prepared them for the COVID-19 pandemic. Sharing current work experience with colleagues through active exchange was perceived as effective and seemed to foster team commitment.

– *Extensive contact with isolated patients even before the pandemic—routine prevents fear!*

*(male*, *35–39 years old*, *prehospital sector)*

– *Teaching units and good exchange among each other during my currently ongoing professional training*. *(male*, *30–34 years old*, *hospital sector)*

– *Link with Italian colleagues to exchange expertise*.

*(male*, *40–44 years old*, *hospital sector)*

– *We had regular team meetings; our superior kept us up to date with all the news*.

*(female*, *50–54 years old*, *outpatient sector)*

One fifth of the reports described early-stage crisis measures as effective in helping crisis prevention. Specifically, the flow of relevant information through internal channels of information including emails or intranet was often mentioned. Nationwide daily information channels such as the Robert Koch Institute (RKI, German Federal Authority for Infectious Diseases) or COVID-19-specific podcasts were also named.

Furthermore, the formulation of clear guidelines and recommendations for action and the timely provision of protective material were both perceived as helpful.

– *Information by my superior*, *information from the clinic management*, *but also passing on information among the assistants/specialists*, *information from senior nurses*.

*(female*, *40–44 years old*, *hospital sector)*

– *Daily "employee news" from the management to the employees in the form of e-mail and information paper at the beginning of the shift*.

*(male*, *30–34 years old*, *hospital sector)*

– *Clear specification of hygiene management in the clinic significantly improved interdisciplinary communication in the clinic and in the department*, *daily updates by the crisis management team of the clinic and the department also by e-mail to my private e-mail address*.

– *Many visual representations of COVID and hygiene procedures by RKI*, *but also on the internet by professional associations or Free Open Access Medical Education*.  *(male*, *60–64 years old*, *hospital sector)*

– *Availability of significantly more protective material*! *Protective measures before and after each patient contact*.

*(male*, *20–24 years old*, *prehospital sector)*

It appears that the perceived effectiveness of crisis prevention varied across sectors. General work experience and exchange were mostly mentioned in the hospital and outpatient sectors, whereas early-stage crisis measures were more often applied in the prehospital sector.

One seventh of the answers were assigned to the category “no effective crisis prevention has been taken” (see S3 Table). Analogous to the acute crisis measures, different reasons for this answer can be derived from the reports: a) preventive measures should have been taken, but they were not, b) no crisis prevention was possible because the situation was unexpected and dynamic, and, c) the measures taken were insufficient. Irrespective of the judgment of how prepared they felt, some healthcare professionals specified that there was d) no specific crisis management or pandemic-specific training. Additionally, some participants perceived no crisis prevention measures on an organizational level but emphasized the use of individual strategies.

*a) None*. […] *Bad (as well as fake) videos of how to put on protective clothing*.

*(male*, *25–29 years old*, *hospital sector)*

*b) None at all*. *Nobody knew how to deal with trainees or how to deal with the training and further education for rescue service personnel*.

*(female*, *35–39 years old*, *prehospital sector)*

*c) None*, *nobody knew about the Coronavirus before*!

*(male*, *35–39 years old*, *hospital sector)*

*d) None*. *Common sense*!

*(male*, *25–39 years old*, *hospital sector)*

#### Individual coping

In the open-ended question about individual coping strategies (which had an overall response rate of 83.4%), three areas were frequently mentioned (which together made up 80% of the given answers, S3 Table): (1) social support, (2) hobbies/leisure activities, and (3) mental strategies. Within social contacts, the family is perceived as mainly supportive, while friends and colleagues were also mentioned as important.

Most of the named hobbies and leisure activities were sport activities. Walks and opportunities to enjoy nature were also frequently reported. Regarding mental strategies, most participants reported the benefits of distractive activities such as watching videos, listening to audio books, social media, or online shopping. In contrast, other respondents mentioned a deliberately reduced media consumption, although these reports were less frequently noted. The use of relaxation/meditation techniques was often reported as a distraction.

Furthermore, a few participants answered with thoughts or plans to quit their job, while others mentioned altruistic or intrinsic motives at work.

– *I don’t enjoy working for the most part for the first time in my life*.

*(male*, *35–39 years old*, *outpatient sector)*

– *I think about changing my job more often*.

*(male*, *40–44 years old*, *prehospital sector)*

– *The desire to help others with it*.

*(female*, *25–29 years old*, *outpatient sector)*

– *Gratitude of the patients*.

*(male*, *30–34 years old*, *outpatient sector)*

## Discussion

### Work stressors

The present study examined different work stressors and their effects on psychological stress among healthcare professionals during the early stage of the COVID-19 pandemic in Germany. Building upon work stressors which have already been discussed in the literature, we identified four underlying latent stress factors: “fear of transmission”, “interference of workload with private life”, “uncertainty/lack of knowledge” and “concerns about the team”. Among these, “interference of workload with private life” was the pivotal predictor for stress responses. Contrary to many assumptions, the factor "concerns about the team” was associated with a lower psychological stress. Items which were originally intended to assess work stressors seem to have a stress-reducing effect. Potentially the factor “concerns about the team” is an indicator of high team commitment and social support by colleagues, which are well-known stress-buffering factors in the literature [39–41]. Alternatively, individuals who experience less stress may be less “self-focused” and may have more developed capacities to be concerned about others. “Fear of transmission” had no effect and “uncertainty/lack of knowledge” had only minor effects on psychological stress. These unexpected findings call for further investigation of work stressor lists among healthcare professionals during the COVID-19 pandemic.

### Psychological stress

In general, the latent stressors had similar effects on psychological stress across work sectors. Healthcare professionals’ stress and fatigue levels during the COVID-19 pandemic were moderate on average, indicating that they were only remotely impacted during the first wave of the pandemic. Reports of high stress levels are a common finding in studies with frontline healthcare professionals during pandemics such as SARS, Ebola [2, 11–13] and more recently during COVID-19, at least among medical staff working in Asia [2, 4, 23, 42] as well as in Europe [15]. High levels of stress were also anticipated for German medical staff [16]. However, Germany was only moderately affected by the pandemic compared to other European countries [5, 43]. The current finding of moderate stress levels among healthcare professionals seems to mirror this impression. An alternative explanation for this effect lies in the structure of the German healthcare system and the prevention measures taken by the German government (e. g., keeping a large proportion of hospital beds free), which proved to be successful [5], as reflected by the moderate stress levels among the examined hospital staff.

There were differences in the stress levels across sectors. The outpatient group was more stressed and less calm, while the prehospital group reported lower fatigue. To better understand these findings, it is necessary to embed them within the specific context of the pandemic situation in Germany. In preparation for the pandemic, considerable burdens on the hospital sector were expected (e.g., scarcity in ICUs; [5]), and so major efforts were targeted at preparing the hospital sector for the pandemic (e.g., clearing wards, generating more ICUs). However, unlike in some neighboring European countries, ICUs in Germany were largely not overcrowded [5] and spare capacities were used to support severely affected neighboring countries. Retrospectively, during the early stage of the COVID-19 pandemic the outpatient sector was affected more severely than the hospital sector, being responsible for screening and testing suspected cases. Health authorities reported and still report that the capacities of outpatient sectors were overstretched in some German regions [44]. Our results mirror this anecdotal impression, since the outpatient group reported that they were more stressed and less calm. This gives reason for concern, since experts expect suspected and infected cases to rise in the flu season, which will again put pressure on outpatient testing resources. While the hospital sector cleared wards to free up ICUs and thereby obviate the anticipated high COVID-19 load, there is currently no possibility of suspending the usual care in the outpatient sector. Given that staff and resources are limited, target-group-specific crisis measures would be helpful to protect healthcare professionals in this sector from severe stress. Thinking beyond upcoming flu seasons, the outpatient sector will also be responsible for additional corona-specific tasks, such as the treatment of an increasing number of patients with "post-COVID-19" complaints and the contribution and administration of future vaccinations. Therefore, in addition to general crisis measures, specifically tailored measures for the outpatient sector should be developed.

Furthermore, most COVID-19 patients received treatment in the outpatient sector in Germany. In addition, rapid expansion of hospital capacities including ICU capacities resulted in larger treatment capacities than required. By contrast, outpatient services could frequently not meet the demands. Therefore, when providing psychological support programs to healthcare professionals, one should also be aware of the high stress burden placed on the outpatient sector.

### Crisis management

The analysis of the open-ended questions substantiates the quantitative findings in the study and highlights the differences between the sectors in terms of their perceived effectiveness of the acute crisis management (crisis measures and crisis prevention). Concerning the crisis measures, the hospital sector profited from organizational measures (e.g., visiting bans or bans on elective surgery), whereas the prehospital sector benefitted from governmental measures (e.g., social distancing and compulsory masks for the general population). The outpatient sector reported that both organizational and governmental measures were effective. Concerning crisis prevention, professional training, work experience and exchange, and crisis measures implemented at an early stage were considered as most important. The hospital and outpatient groups benefitted from experience and exchange, while the prehospital sector profited from crisis measures implemented at an early stage. In sum, specific early crisis measures on the governmental level, together with later organizational measures, were successful in reducing healthcare professionals’ stress, and thereby contributed to the protection of healthcare professionals and their mental health, well-being, and functioning.

**Fig 2:**
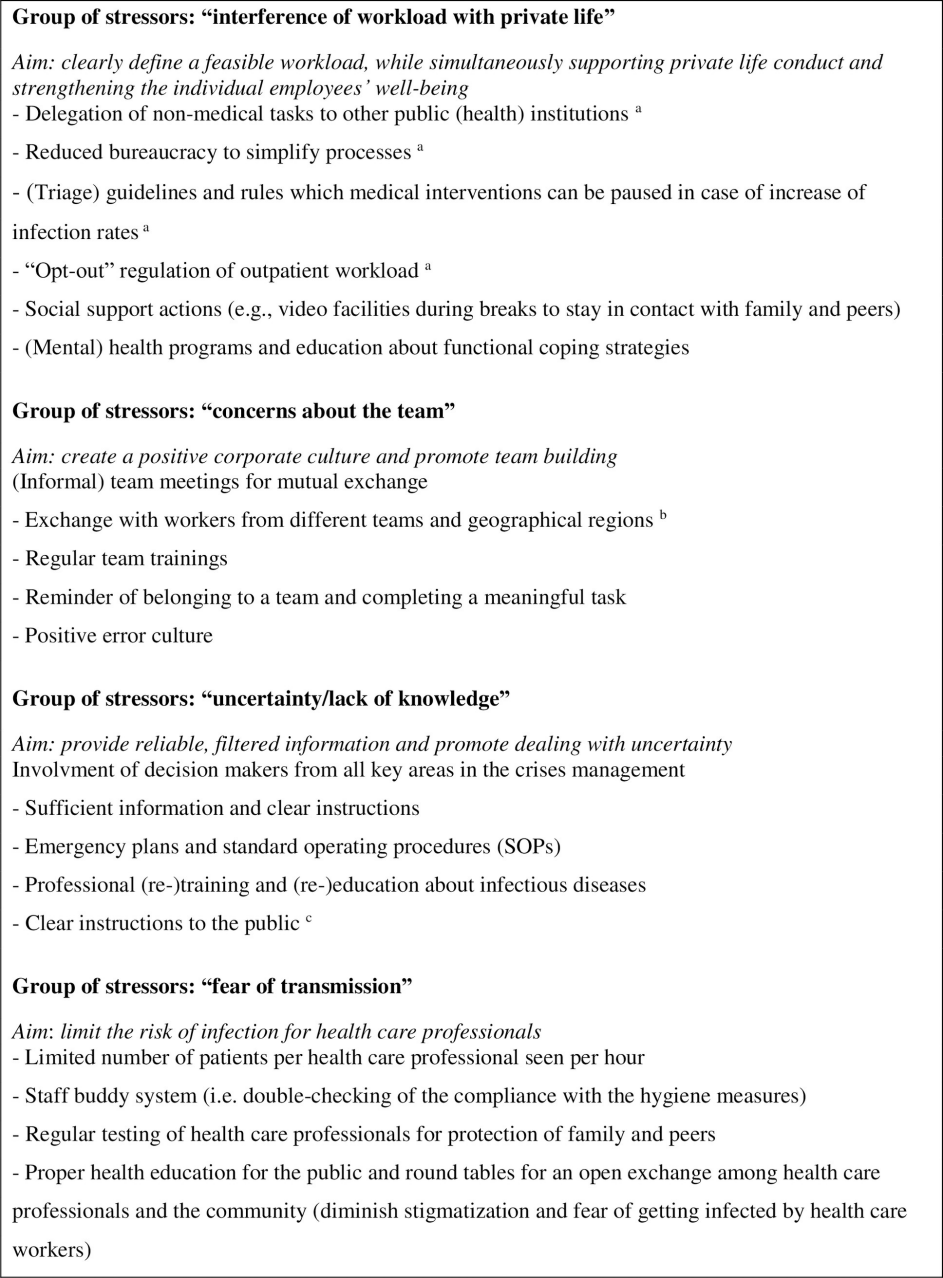
Recommendations to strengthen coping resources in health care professionals. Note. ^a^ special recommendation for the outpatient sector; ^b^ special recommendation for the hospital sector; ^c^ special recommendation for the prehospital sector.

From the results of this study, decision makers in the healthcare sectors should take away two central messages: 1) there should be a priority focus on work stressors related to “interference of workload with private life” in all sectors; and 2) “concerns about the team” potentially driven by high team commitment should be used as a work-specific coping resource that reduces stress responses and improves mental health, well-being, and functioning. In the following, the four latent work stressors, their influence on healthcare professionals’ psychological stress and the distinctive relationships between the sectors are discussed. Finally, specific recommendations for effective crisis management (Fig 2) will be derived for each factor and embedded into the existing research.

**“Interference of workload with private life”** comprises the higher volume of work, which results in difficulties and reconciliation of work and family as well as insufficient capacities for stress regulation, leading individuals to seek social support or basic self-care. In order to better deal with this work stressor, decision makers should clearly define a feasible workload, while simultaneously supporting private life conduct and strengthening the individual employee’s well-being.

Based on the premise that only mentally healthy employees are able to constantly perform at a high level [45, 46], decision makers should implement several steps to limit workload in the context of pandemics. It is essential for healthcare professionals to have enough resting hours at home, appropriate working shifts, and regular breaks [2] in order to enable flexible and family-friendly working hours. There is also a need for preventive concepts for highly affected employees [14]. Furthermore, employers are advised to ensure resting possibilities and opportunities to engage in positive coping strategies during work [26]. With regard to mental health in particular, higher authorities should provide appreciation and positive feedback to employees for their work [14, 43]. Especially in the event that, due to possible staff absences, organizational measures such as sufficient breaks may temporarily not be adhered to, it is important to signal an awareness of this exceptional situation to employees and to appreciate their work so as to strengthen their mood and motivation.

Given that healthcare professionals in the outpatient sector seemed to be more affected by the pandemic, specific measures regarding the organization of the outpatient sector in Germany are proposed to reduce their workload. Non-medical tasks should be delegated to other public health institutions, e.g., the supply of protective material, coordination of testing strategy, clear rules of conduct for certificates, and uniform remuneration for COVID-19 services. Processes can be accelerated by reducing bureaucracy, e.g., simplifying the accounting of COVID-19 services. Further preventive measures should include an “opt-out”-regulation of the outpatient workload, in order to avoid compulsory assignment to caring for patients with COVID-19 [2]. Therefore, (triage) guidelines and rules which note the medical interventions that can be skipped or paused in the outpatient sector in the case of increasing COVID-19 cases should be developed as soon as possible.

In times of increased workload, social support from family and friends can buffer against psychological stress [2]. Decision makers are responsible for taking various measures to enable social support from family and friends at the workplace, as employees spend an especially large part of their time there during this crisis and are in need of sufficient social support to be able to fulfill their duties and look after their mental health. Social support can be provided by organizational measures, e.g., through the establishment of video facilities for staff during breaks to maintain contact with families and alleviate their concerns [2]. Establishing psychological interventions and education on healthy coping strategies can strengthen well-being, resilience and self-regulation strategies [2, 8]. Low-threshold psycho-social help should also be offered to the healthcare professionals [2, 14]. As an example for best practices, a psycho-social emergency care (“Psychosoziale Notfallversorgung”: PSNV) has been established in Germany. This program assesses concerns and needs, practical support and care, empathic listening, access to information, services and social support, and protection from further harm [14]. Expanding this concept, psycho-social help may also be offered to healthcare professionals’ relatives to support them in supporting the professionals. Nevertheless, the answers to the open-ended questions suggest that healthcare professionals already know stress regulation strategies that work for them. Healthcare professionals reported that the reinforcement of earlier functional coping strategies, such as hobbies and leisure activities or certain adaptive mental strategies (e.g., the use of relaxation or meditation techniques, reduced media consumption or the visualization of reasons to work on the frontline) were effective. Since (mental) health programs and effective individual coping strategies seem to be more effective when ritualized, these measures should be constantly cultivated, especially in non-pandemic times [14].

**“Concerns about the team”** includes concerns about isolation from colleagues, passing the virus to the workplace and heavier workload for colleagues when falling ill. Interestingly, “concerns about the team” reduced healthcare professionals’ stress responses, indicating that caring for colleagues does not represent a stressor but rather a protective factor. At first glance, this finding may call into question the validity of the work stressors that were empirically tested. However, in interpreting “concerns about the team” as an indicator for high team commitment, decision makers can make use of the protective impact of this factor. In order to better deal with "concerns about the team”, decision makers should pursue the aim of creating a positive corporate culture and promote team building.

This quantitative finding is substantiated by the open answers which suggested that sharing (current) work experience with other frontline healthcare professionals was perceived as a pivotal support. Active exchange with colleagues in their own institution, the same region, nationally and internationally made them feel better about working under pandemic circumstances and boosted their confidence at work. Regular team trainings as well as informal team meetings for mutual exchange and a positive safety culture are strategies which build team commitment and offer a conscious reminder of belonging to a team and completing a meaningful task [14]. Through the concept of assertive communication, teams can learn to engage in a more fluid, frank, and direct communication among their members [41].

Given the beneficial influence of “concerns about the team” on stress responses, this finding hints at higher team commitment in the prehospital sector, protecting them from negative health consequences of stress. Therefore, it might be useful to identify team building measures in this sector and to learn from them. At the same time, the qualitative data show that the hospital and the outpatient sector had a higher tendency for exchange between workers from different teams and regions. The lesson learned from this fruitful exchange in the hospital and the outpatient sector is that specific platforms should be created and promoted for all three sectors in order to enable people who have experience in treating similar diseases or situations to train, lead or support the teams who do not have such experiences.

**“Uncertainty/lack of knowledge”** describes the uncertainty in action caused by an information load of constantly changing information, lack of clear instructions and insufficient information about the long-term health consequences of COVID-19. Other facets of this stressor include being insufficiently supplied with personal protective equipment and strict bio-security measures. These facets have also emerged in other work contexts during the COVID-19 pandemic [26]. In order to cope with this work stressor, decision makers should pursue the aim of providing reliable, filtered information and the aim of developing programs to train dealing with uncertainty.

For clear communication among healthcare professionals, the subjective reports in our study advocate the involvement of decision makers from all key areas in the crisis management. Knowledge should be shared in regular meetings of representatives for all relevant local public health authorities (e.g., public health department, general practitioners and pediatricians, hospital providers, laboratory managers, and public order department). In this process, a clear distribution of roles and tasks and its constant adaptation, where necessary, might ease communication processes [14]. Based on the information gathered in these meetings, emergency plans and standard operating procedures (SOPs) should be mutually developed [2, 12]. SOPs represent a set of step-by-step instructions, which are designed to help workers carry out complex routine operations. They aim at optimizing efficiency, quality output and performance, while at the same time reducing miscommunication. In future, clear communication structures within these authorities are necessary to make sure that the target-specific information outcomes are transferred to frontline healthcare professionals. In line with previous studies [2, 14], regular professional training and (re-)education about infectious diseases were perceived as particularly effective.

**“Fear of transmission”** mainly captures the fear of infecting others, such as friends, families and colleagues, and the worries about vulnerable family members and friends which is in line with previous work [13, 23]. It also includes the participants’ fear of becoming infected themselves.

In order to meet the stressor "fear of transmission”, decision makers should pursue the aim of limiting the risk of infection for healthcare professionals, which in turn will limit the risk of transmission. Limiting the risk of infection for healthcare professionals can be achieved in several ways. One effective way is to provide enough protective clothing so that healthcare professionals are protected when caring for infected patients. Bearing in mind that protective clothing has been scarce at the beginning of the pandemic, which resulted in healthcare professionals having to wear protective clothing designed for one-time-use several times, this way of limiting the risk of infection is crucial.

Further, in promoting individual responsibility, healthcare professionals should have easy access to reliable, filtered information and regular hygienic (re-)training. Beyond taking responsibility for oneself, a staff buddy system (which involves double-checking the precise compliance with the hygiene measures by a colleague) is proposed in the literature [2]. Limiting the number of patients who are seen by one healthcare professional per hour may be an additional preventive measure.

Potentially, “fear of transmission” is triggered by the stigmatization and exclusion from public life that healthcare professionals had to face during the first wave of the pandemic. Consequently, proper health education for the public and round tables which allow open exchange among healthcare professionals and the community can help to prevent misinformation and stigmatization and, in turn, reduce healthcare professionals’ fear of transmission [47, 48].

### Strengths, limitations and future directions

Drawing on existing recommendations concerning healthcare professionals’ work stressors during a pandemic [14, 19–21], our study is one of the first empirical investigations of these work stressors during the COVID-19 pandemic in Germany. While there are early studies conducted in Asia on healthcare professionals’ stress responses to the COVID-19 pandemic which concentrate on the outpatient sector [4], the present study included diverse subsamples from all healthcare sectors, including outpatient, prehospital and hospital sectors. The use of open-ended questions enabled us to provide a context to the quantitative analyses, resulting in a more in-depth understanding of the impact of the COVID-19 pandemic on healthcare professionals’ psychological stress experiences. A further advantage of the qualitative supplementary data is that, additionally to a deeper understanding of the individuals themselves, their context and life circumstances can be further investigated. This information is of particular importance for understanding the four main stressors examined in the present study since all four of them relate to the individual’s living environment. Hence, information concerning this environment is particularly useful.

At the same time, some limitations apply to the present study. First, the minority of participants (less than 6%) took part in more than one measurement occasion, thereby delivering no truly longitudinal information on intra-individual change. Therefore, the current results should be interpreted as cross-sectional. To increase sample sizes, we distributed the online survey widely across various channels, which reduced the control of the size and demographic characteristics in each subsample. As a consequence, the geographic distribution across Germany and (work) contexts might differ strongly between participants. Medical doctors are also overrepresented in the current sample, which might limit the generalizability to other healthcare professionals. To ensure high participation rates and to keep interference with professional duties to a minimum, we used questionnaires which were originally developed for ecological momentary assessment. Thus, the findings might be limited by the use of single-item measures. Caution must also be taken in the interpretation of the qualitative data: while the response rate for personal helpful coping strategies was high (83.4%), only half of the participants responded to the questions about effective crisis measures and crisis prevention (see also [4]).

Having acknowledged the strengths and limitations of the study, its results offer interesting directions for future research. First, the study captured a two-month period at the beginning of the pandemic. Given that epidemiologists already predict further waves, healthcare professionals might continue to be confronted with work stressors related to the COVID-19 pandemic. Our results suggest that, even in the early stage of the pandemic, the perceived severity and impact of the stressors are dynamic. The present study starts at a very early stage of the pandemic when there is virtually no previous experience in dealing with such a situation, enabling it to filter out the true stressors, since no structures have yet been created to facilitate and systematically deal with the situation. The study thus represents a valid starting point for future investigations with regard to stressors. During further outbreaks, it would be interesting to investigate how the four stressors are perceived at a similarly acute time-point, whether new stressors appeared or old ones disappeared and, ultimately, whether following the suggested action guidelines has an impact on the situation at a similar point of time during further waves. Hence, it is necessary to monitor the long-term impact of the pandemic and its related stressors, as chronic stress can have tremendous health consequences [49]. Second, the present study proposes several implications and crisis measures for successfully coping with a pandemic. Future studies should test the efficacy of these measures. Previous studies in healthcare settings [50] and during the pandemic [26] have shown that the use of emotion regulation strategies can help individuals to cope successfully with work stressors and reduce stress responses. While the focus of the present study lays on the identification of stressors, future studies should examine how mechanisms of stress regulation apply to long-lasting, exceptional stress situations such as those experienced during a pandemic.

## Conclusion

The present study identified four latent work stressors among German healthcare professionals during the COVID-19 pandemic. “Interference of workload with private life” was the pivotal predictor of stress responses, whereas “concerns about the team” had stress-buffering effects. Our findings suggest that the outpatient sector has been affected more severely than the well-prepared hospital sector, since its capacities have been overstretched. In light of a predicted increase to COVID-19 patient load and vaccination duties, specific measures should now be taken to prepare the outpatient sector for the future. To meet the work stressors, healthcare professionals need sector-specific psycho-social support within and outside the workplace that reduces their stress responses and protects their mental health, well-being, and functioning. At the workplace, “concerns about the team” buffers against adverse stress responses, although it involves being more stressed about colleagues’ well-being. As healthcare professionals are predicted to continue to deal with stress as a result of the pandemic in the upcoming months, it is important for decision makers to reduce stress as much as possible, and for healthcare professionals to identify and enforce individual positive coping strategies.

## Supporting information

S1 Table Factor correlations. (DOCX)

S2 Table Four-factor solution form the exploratory factor analysis on work-related stressors: Pattern matrix and structure matrix. (DOCX)

S3 Table Frequencies of personal coping strategies, effective crisis measures and effective crisis prevention across sectors (in % of all coded answers). (DOCX)
