# Supplementary material for: Stressors faced by healthcare professionals and coping strategies during the early stage of the COVID-19 pandemic in Germany
Source: PLoS One. 2022 Jan 18;17(1):e0261502. doi: 10.1371/journal.pone.0261502 (PMC8765664; doi:10.1371/journal.pone.0261502)
Supplement: S1 Table — (DOCX) [file pone.0261502.s001.docx]

**S1 Table**. Factor correlations.

|  | Factor 1:  “fear of transmission” | Factor 2:  “interference of workload with private life” | Factor 3:  “uncertainty/lack of knowledge” | Factor 4:  “concerns about the team” |
| --- | --- | --- | --- | --- |
| Factor 1:  “fear of transmission” | - |  |  |  |
| Factor 2:  “interference of workload with private life” | .53** | - |  |  |
| Factor 3:  “uncertainty/lack of knowledge” | .48*** | .59*** | - |  |
| Factor 4:  “concerns about the team” | .19 | .15 | .23 | - |

Note. *** *p* < .001, ***p* < .01.
