# Supplementary material for: Stressors faced by healthcare professionals and coping strategies during the early stage of the COVID-19 pandemic in Germany
Source: PLoS One. 2022 Jan 18;17(1):e0261502. doi: 10.1371/journal.pone.0261502 (PMC8765664; doi:10.1371/journal.pone.0261502)
Supplement: S2 Table — (DOCX) [file pone.0261502.s002.docx]

**S2 Table**. Four-factor solution form the exploratory factor analysis on work-related stressors: pattern matrix and structure matrix.

|  |  | Factor 1:  “fear of transmission” | | Factor 2:  “interference of workload with private life” | | Factor 3:  “uncertainty/lack of knowledge” | Factor 4:  “concerns about the team” |
| --- | --- | --- | --- | --- | --- | --- | --- |
|  | Work-related stressor | | Pattern Matrix (Geomin-Rotated Loadings)/  Structure Matrix *(*Item-Factor Correlations*)* | | | | |
| 1 | “Fear of getting infected” | | 0.521 / *0.597* | | 0.034 / *0.365* | 0.055 / *0.363* | 0.165 / *0.283* |
| 2 | “Fear of infecting others” | | **0.898** / ***0.826*** | | -.124 / *0.334* | -.034 / *0.337* | 0.047 / *0.192* |
| 3 | “Worrying about family members/children being at home” | | 0.480 **/** *0.579* | | 0.271 / *0.489* | 0.005 / *0.341* | -.238 / *-.103* |
| 4 | “Worries about health deteriorations of vulnerable family members and friends” | | 0.664 / *0.717* | | 0.163 / *0.485* | -.003 / *0.376* | -.161 / *-.009* |
| 5 | “Stigmatization” | | 0.139 / *0.438* | | 0.482 / *0.584* | -.040 / *0.387* | **0.339** / ***0.430*** |
| 6 | “Strict bio-security measures” | | 0.026 / *0.312* | | 0.378 / *0.499* | 0.188 / *0.419* | -.012 / *0.094* |
| 7 | “Higher work demands” | | 0.117 / *0.426* | | 0.599 / *0.653* | 0.018 / *0.394* | 0.017 / *0.128* |
| 8 | “Reduced capacity to use social support” | | -.060 / *0.398* | | **0.826** / ***0.808*** | -.030 / *0.471* | 0.199 / *0.308* |
| 9 | “Insufficient capacity to implement basic self-care” | | -.004 / *0.406* | | **0.734** / ***0.770*** | 0.090 / *0.496* | -.099 / *0.033* |
| 10 | “Insufficient information about long-term exposure to infected individuals” | | 0.297 / *0.586* | | 0.197 / *0.570* | 0.339 / *0.624* | 0.117 / *0.281* |
| 11 | “Fear of infecting friends and family” | | **0.881** / ***0.882*** | | 0.005 / *0.469* | 0.016 / *0.433* | -.048 / *0.126* |
| 12 | “Confrontation with anger against the government by patients” | | 0.096 / *0.408* | | 0.379 / *0.568* | 0.238 / *0.505* | -.009 / *0.122* |
| 13 | “Information overload of constantly changing information” | | -.071 / *0.314* | | 0.072 / *0.462* | **0.740** / ***0.737*** | -.050 / *0.116* |
| 14 | “No clear instructions” | | 0.038 / *0.449* | | -.055 / *0.499* | **0.909** / ***0.897*** | 0.010 / *0.215* |
| 15 | “Fear of being isolated from usual work team” | | -.029 / *0.324* | | 0.413 */ 0.539* | 0.160 / *0.459* | **0.311** / *0.405* |
| 16 | “Worries about heavier workload of coworkers when falling ill” | | 0.255 / *0.480* | | 0.292 / *0.492* | 0.047 / *0.399* | 0.253 / *0.358* |
| 17 | “Insufficient protective clothing” | | 0.191 / *0.417* | | 0.030 / *0.378* | 0.399 / *0.529* | 0.091 / *0.223* |
| 18 | “Difficult reconciliation of work and family” | | 0.166 / *0.500* | | 0.531 / *0.689* | 0.130 / *0.513* | -.035 / *0.107* |
| 19 | “Fear of passing virus to workplace” | | 0.529 / *0.616* | | -.003 / *0.358* | 0.065 / *0.385* | 0.296 / ***0.412*** |

Results obtained from Robust-ML Exploratory Factor Analyses ran with Mplus 8.1 (Muthén & Muthén, 2017). Table cell entries: Geomin-rotated loadings (printed upright) and zero-order correlations of the item with the factor (slanted). Boldfaced values show the two highest loadings or correlations per factor.
