# Supplementary material for: Stressors faced by healthcare professionals and coping strategies during the early stage of the COVID-19 pandemic in Germany
Source: PLoS One. 2022 Jan 18;17(1):e0261502. doi: 10.1371/journal.pone.0261502 (PMC8765664; doi:10.1371/journal.pone.0261502)
Supplement: S3 Table — (DOCX) [file pone.0261502.s003.docx]

**S3 Table.** Frequencies of personal coping strategies, effective crisis measures and effective crisis prevention across sectors (in % of all coded answers).

|  | | Sectors | | | |  | |  | |
| --- | --- | --- | --- | --- | --- | --- | --- | --- | --- |
|  | Hospital sector | | Prehospital  sector | | Outpatient sector | | NAs^*^ | | Total |
| **Private stressors** Caregiving duties Economic and domestic obligations Health concerns  Romantic relationships Social distancing Worries about relatives High workload Others **Personal coping strategies** | | **17** 10 **13** 8 8 **31** 8 5 | | 6 **14** 5 **14** 12 **29** 11 10 | **22** 10 10 **15** 5 **32** 5 2 | 0 0 0 0 0 **100** 0 0 | | **13** **12** 9 **12** 9 **31** 9 6 | |
| Social contacts | | **41** | | **38** | **37** | **60** | | **39** | |
| Hobbies/leisure activities | | **26** | | **23** | **17** | **40** | | **23** | |
| Mental strategies | | **15** | | **16** | **24** | 0 | | **17** | |
| Well-considered use of media/information | | 8 | | 9 | 6 | 0 | | 8 | |
| Work-related strategies | | 2 | | 5 | 4 | 0 | | 4 | |
| Consideration of basic needs | | 1 | | 2 | 1 | 0 | | 1 | |
| Miscellaneous | | 7 | | 6 | 10 | 0 | | 7 | |
| **Effective crisis measures** | |  | |  |  |  | |  | |
| Governmental measures | | **36** | | **45** | **33** | **33** | | **39** | |
| Organizational measures | | **46** | | **23** | **35** | **33** | | **35** | |
| None | | **15** | | **25** | **23** | 0 | | **20** | |
| Not applicable | | 3 | | 7 | 10 | **33** | | 6 | |
| **Effective crisis prevention** | |  | |  |  |  | |  | |
| Training | | **24** | | **25** | 14 | **50** | | **23** | |
| Self-study | | **15** | | 12 | 14 | 0 | | 13 | |
| Experience | | **28** | | **16** | **29** | **50** | | **23** | |
| Preventive crisis measures | | 14 | | **30** | **15** | 0 | | **21** | |
| None | | 13 | | 13 | **20** | 0 | | 14 | |
| Not applicable | | 6 | | 3 | 8 | 0 | | 5 | |

Note. Numbers in bold represent the three most frequently mentioned categories. *Values in this column refer to answers from participants who did not disclose the sector they were working in.
